# Development and testing of a summative video-based e-examination in relation to an OSCE for measuring communication-related factual and procedural knowledge of medical students

**DOI:** 10.3205/zma001466

**Published:** 2021-03-15

**Authors:** Stephanie Ludwig, Lina Behling, Uwe Schmidt, Sabine Fischbeck

**Affiliations:** 1 University Medical Center, Johannes Gutenberg University Mainz, Clinic and Polyclinic for Psychosomatic Medicine and Psychotherapy, Department of Medical Psychology and Medical Sociology, Mainz, Germany; 2 Johannes Gutenberg University Mainz, Center for Quality Assurance and Development (ZQ), Mainz, Germany

**Keywords:** medical education, communicative competencies, summative assessment, video-based examination, OSCE

## Abstract

**Objective: **In the context of educating medical students, testing of competence in medical communication is carried out primarily with the Objective Structured Clinical Examination [1]. This makes it possible to assess practical performance, but it is resource-intensive and has a negative impact on test quality. The project “Digital test tool for measuring communication skills in medical studies” (digiRole) was funded by the BMBF (Federal Ministry of Education and Research) and its objective was to develop digital formats as electronic versions of an OSCE in order to test the communication competency of medical students. Such digital forms of examination should be cost-effective, be relevant to clinical practice and have high psychometric quality. In terms of content, the examination questions should incorporate factual and procedural knowledge as components of communication competency, although we assumed that procedural knowledge is more relevant than facutal knowledge to OSCE performance. This article describes the development and testing of a video-based, communication-related e-examination that is relevant to passing the test, which is the first milestone of the overall project.

**Methodology: **We produced videos and related exam questions in the form of a situational judgement test [2] related to medical psychology and medical sociology, based on the educational content of a preclinical course on doctor-patient communication at the Mainz University Medical Center. In the summer semester of 2018, 226 students sat for this video-based single-choice e-examination (VSE). In the winter semester of 2018/2019, a different cohort of 192 students participated in the VSE as well as a tried-and-tested communication OSCE with five stations [3].

**Results: **The internal consistencies for the VSE in the summer semester of 2018 were α=.55, in the winter semester 2018/19 with α=.62 and for the OSCE with α=.60. There was a positive correlation between the performance of the students with the VSE and that with the OSCE (r=.21, p≤.01). Principal Axis Analyses did not reveal any dimensioning in terms of factual and procedural knowledge. In the evaluation, the majority of the students stated that the VSE was quite relevant to the practise of medical communication and were in favour of retaining this form of examination.

**Conclusion: **The correlation between the VSE and the OSCE is relatively low, so that the VSE in this form is not a satisfactory predictor of an OSCE result. In terms of internal consistency, the VSE and the OSCE produced an almost identical result. It can also be assumed that the VSE can achieve a high degree of objectivity with the use of standardised video-based examinations as well as greater resource efficiency than OSCEs.

## Zusammenfassung

**Zielsetzung:** Die Überprüfung ärztlicher kommunikativer Kompetenzen erfolgt im Rahmen des Medizinstudiums primär über die Prüfungsform OSCE [1]. Diese ermöglicht eine Bewertung praktischer Leistungen, ist allerdings ressourcenaufwändig und bringt Limitationen hinsichtlich der Testgüte mit sich. Die Zielsetzung des vom BMBF geförderten Projekts „Digitales Prüfungstool zur Messung kommunikativer Fertigkeiten im Medizinstudium“ (digiRole) war daher die Entwicklung digitaler Formate von Prüfungen kommunikativer Kompetenzen von Medizinstudierenden als elektronisch gestützte Varianten einer OSCE. Die entsprechenden Prüfungsformen sollten dabei ökonomisch sein, einen hohen Praxisbezug herstellen und eine hohe psychometrische Qualität aufweisen. Inhaltlich sollten die Prüfungsfragen Fakten- und Handlungswissen als Bestandteile der kommunikativen Kompetenz abbilden, wobei davon ausgegangen wurde, dass Handlungswissen stärker mit der OSCE-Leistung zusammenhängt als Faktenwissen. Der vorliegende Artikel beschreibt die Entwicklung und Erprobung einer bestehensrelevanten videobasierten kommunikationsbezogenen E-Prüfung als ersten Meilenstein des Gesamtprojekts.

**Methodik: **Auf Grundlage der Lerninhalte eines vorklinischen Kursus zur Arzt-Patient-Kommunikation der Universitätsmedizin Mainz im Fach Medizinische Psychologie und Medizinische Soziologie wurden Videos produziert und dazu in Bezug stehende Prüfungsfragen mit Aufgaben im Sinne eines Situational Judgement Tests [2] konzipiert. Im Sommersemester 2018 nahmen 226 Studierende an dieser videobasierten Single-Choice Prüfung (Video-based Single-Choice Examination, VSE) und im Wintersemester 2018/2019 192 Studierende (unterschiedliche Kohorten) an der VSE sowie an einer bereits gut erprobten Kommunikations-OSCE mit fünf Stationen teil [3].

**Ergebnisse: **Die internen Konsistenzen lagen für die VSE im SS 2018 bei α=.55, im WS 2018/19 bei α=.62 und für die OSCE bei α=.60. Die Leistungen der Studierenden in VSE und OSCE waren positiv korreliert (r=.21, p≤.01). Hauptachsenanalysen ergaben keine Dimensionierung in die Aspekte Fakten- und Handlungswissen. Die Studierenden gaben in der Evaluation mehrheitlich an, dass in der VSE der Bezug zur Praxis des ärztlichen Gesprächs gut hergestellt wird und sprachen sich für eine Beibehaltung dieser Prüfungsform aus.

**Schlussfolgerung: **Der Zusammenhang zwischen VSE und OSCE fällt relativ gering aus, so dass die VSE in dieser Form noch nicht hinreichend ein OSCE-Ergebnis prädizieren kann. Hinsichtlich der internen Konsistenz konnten die VSE und die OSCE ein nahezu identisches Ergebnis erzielen. Es kann des Weiteren angenommen werden, dass die VSE durch den Einsatz von standardisierten videobasierten Prüfungsaufgaben einen hohen Grad der Objektivität sowie ein im Vergleich zu OSCEs ressourcenschonenderes Vorgehen erzielen kann.

## 1. Introduction

### 1.1. State of research

Teaching and testing communication skills is becoming increasingly relevant in the context of studying medicine. The “Master Plan for Medical Education 2020” emphasises the importance of relevant training as part of the medical course [4]. Education in communication skills is recognised internationally as an essential part of medical training [5], [6]. With regard to styles of teaching and examination relevant to competence in medical communication, Härtl et al. [1] surveyed universities in German-speaking countries and found that teachers use models or catalogues of learning objectives in 70% of degree programmes. The concept of competence in medical communication is regarded as complex and multi-layered [7], [8]. The review article [1] for the German-speaking area and one by Laidlaw et al. [9] for Great Britain also show that, with regard to the types of examination, testing competence in communication takes place primarily in the form of the practical OSCE (Objective Structured Clinical Examination). The written test is the second most common form in German-speaking countries. In other countries such as Australia, Canada and the USA, the OSCE style of examination is also used in addition to classic written examinations [10], [11], [12]. With the OSCE, the student’s competence is assessed by the examiners, who mostly use checklists based on simulated situations at several examination stations - sometimes with the aid of simulated patients. When comparing written forms of examination, the advantage of an OSCE is its high practical relevance and the possibility of a performance test at a behavioural level [13]. By contrast, written exams are at the cognitive level, and can be broken down into factual knowledge and procedural knowledge [13]. This level can be checked, for example, with the aid of single or multiple choice questions, key feature concepts (making critical decisions) or situational judgement tests [2], [14], [15], [16]. In a situational judgement test, scenarios from professional practice are presented in writing or video-based. An appropriate action must be decided in the context of the examination questions. The response can be formulated in various ways. The internal consistencies are mostly good [2]. Situational judgement tests are already used in medical training [15]. In terms of economy, implementation of an OSCE is very time consuming and resource-intensive with regard to financial and personnel aspects. The reliability and content validity of an OSCE is largely determined by the number of stations [17], which makes the effort enormous and often unaffordable in the desired form. Assessment by various auditors may provide different results, so that inter-rater reliability (and therefore objectivity) may be inadequate [17]. However, a written test (such as a situational judgement test) generally achieves greater objectivity and reliability due to the standardisation of questions and evaluation as well as a greater number of questions.

The presented advantages and disadvantages of the two most common ways of examining competence in medical communication raise the question of alternative forms of examination that take into account the importance of a high practical relevance as well as the requirements of a realistic resource procedure with a high test quality. Digital formats with videos appear to be ideal in the field of communication competence, since they enable case-based testing with a high degree of practical relevance and a large number of students. This is consistent with student feedback from the classroom that doctor-patient consultations shown in videos are much closer to a real-life situation than just written case descriptions, in which images, sound, facial expressions and gestures are completely missing. This makes it easier for students to imagine themselves in the role of a doctor and “experience” the situation than it would be with just written exams. There is also the option of incorporating questions and videos into exam software, so that the examination can be designed economically and objectively. Application of digital formats to teaching competence in medical communication is familiar to the authors, mainly in relation to video-based exercises for learning about competence in communication when this project started in German-speaking areas in 2017 [18]. With regard to examinations, the only report of the use of videos in examinations (at two universities) is that of Härtl et al. [1]. Several relevant studies have been carried internationally that deal with the development and testing of video-based examinations for measuring skills in medical communication [8], [19], [20]. At universities where these studies were carried out, an Objective Structured Video Exam (OSVE) was used, in which students were given tasks related to videos on doctor-patient consultations, which included questions in short essay format and naming communication skills. Humphris and Kaney [19] found a correlation of r=.17 between the OSVE and the OSCE.

#### 1.2. Project design

The lack of resource-saving, competence-oriented exams with significant practical relevance led us to the idea of developing, testing and scientifically investigating a video-based examination of factual and procedural knowledge as components of communication skills in medical studies for the first time in German-speaking regions. The exam should be regarded as a digital variant of the OSCE. In the first phase of the study, the video-based examination was designed as a summative examination (video-based single-choice examination, VSE) with tasks in the form of a situational judgement test. For the reasons outlined above, we assume that the VSE can achieve higher practical relevance than purely written examinations (also felt by the students), proves to be economical, and achieves a high level of implementation and objectivity of evaluation (with software management). The high level of economy is expected because, although much time and money need to be invested in creating the scenarios and producing the videos, there should be enough material to enable students to be tested inexpensively over several semesters. This makes it possible to reduce the amount of time taken by the examiners with the OSCE. It is also assumed that there is a positive correlation between the examination performance of the students with the VSE and their performance with the OSCE (cf. also [19] and [21] with r=.32 for the correlation of key feature testing and the OSCE). The underlying dimensions of the VSEs will be examined, and it is conceivable that the design of the examination is reflected in a two-factor solution across all content-related topics in accordance with the two aspects “factual knowledge” and “procedural knowledge”. Should such a solution arise, one should also find out whether the questions in the VSE at the level of procedural knowledge are more closely related to the results of the OSCE than the questions on the level of factual knowledge, since it is obvious that procedural knowledge and the action taken seems to be more closely related than factual knowledge and the action taken. All examinations should be evaluated by the students in order to assess their practical relevance and whether or not this form of examination should be maintained.

## 2. Methods

### 2.1. Study participants and process

A VSE was used for the first time in the course II "Doctor-Patient Communication" for students studying medicine in the second semester at the Mainz University Medical Center in the summer semester of 2018 (SS18). In the winter semester of 2018/19 (WS18/19), the students of the following cohort in the aforementioned course in the second semester also completed the VSE and then the OSCE. Prior to implementation, the courses included explanations of the precise design of the exams with a sample task. There was also a detailed information sheet that could be read separately. The VSEs were carried out on the premises of the Centre for Data Processing at the University of Mainz at the end of the semester. The technical implementation took place with ILIAS testing software. Except for a few problems with the sound quality and the loading times of the videos, which were easily resolved, implementation of the VSEs ran smoothly without any problems. After the examination, the evaluation was carried out with paper-and-pencil surveys. The higher response rate in the evaluation of the VSE in the second cohort is probably due to the fact that the examination supervisors referred more to evaluation based on experiences from the previous semester and collected it in a targeted manner. Information on the number, age and gender of the study participants is given in table 1 (Tab. 1).

#### 2.2. Development of the VSE

In the development phase of the VSE, which was based on the examination content from previous years, case reports and dialogues between doctor and patient were developed in an initial step. The dialogues contain the essential theoretical concepts that students should be able to apply in practice (including the Calgary-Cambridge scheme [22], NURSE model [23], counselling techniques such as active listening [24], principles of participatory decision-making [25] and the SPIKES protocol [26]). The scenarios incorporate the following requirements: checking and promoting drug compliance, discussing medical histories, communicating a cancer diagnosis according to the SPIKES protocol, conducting ward rounds, discussing therapy for hypertension according to the principle of participatory decision-making with the patient, changing the behaviour of obese patients, analysing stress reactions and conveying information. The dialogues were checked and revised by course instructors. Interviews with experts (including general practitioners and psychologists) were also carried out to ensure authenticity. Experts were selected on the basis that they had practical experience in everyday working life, were perhaps involved in teaching themselves or, in one case, had been involved in the development of a previous OSCE. The experts gave written feedback on the dialogues (based on the questions asked, e.g. requesting an assessment of the credibility of the setting, medical history, conducting a consultation, presenting a patient etc.). These were evaluated qualitatively and the dialogues were revised accordingly.

The videos were produced in collaboration with Mainz University’s media centre and the learning clinic of Mainz University’s Medical Center. Simulated patients and a general practitioner took the appropriate roles. A split-screen process was used to process the video content (both long and frontal shots) so that assistance with changing perspectives could be avoided. Consistent with the recommendation of Hoppe-Seyler et al. [27], the students opinion on the credibility of the shown scenes was regarded as important, therefore a trial production of a scene was made prior to the final filming. This was evaluated by eight students (selected from an e-mail distribution list for medical students from different semesters). The “doctor” role (63%), “patient” role (75%), “practice” (88%) and “interaction” (88%) were assessed by the majority of the students as realistic.

The questions related to the exam videos were video-adapted and developed in a single-choice format, each with five answer options and reference to existing exam questions. The tasks require either factual knowledge of the presented sequence or a practical decision/choice of a suitable formulation in the sense of a situational judgement test. This procedure takes into account the course design with theoretical and practical content. In addition, it was decided to question the wide range of topics covered in the course and not to narrow it down to specific topics. In the first exam, the focus was slightly more factual knowledge and in the second exam slightly more on procedural knowledge (for the breakdown into factual and procedural knowledge, see table 2 (Tab. 2)). The questions were checked and revised by course instructors.

The examination tasks were held in the following style. The video shows the case of a 50-year-old patient who was diagnosed with hypertension and for whom a treatment decision must be made according to the principle of participatory decision-making. The video shows how the female doctor tells the patient that it is important to her to make a decision about the treatment steps together with him, and the patient agrees. The subsequent task reads “The doctor and patient have now agreed on equal rights with regard to the decision. When implementing participatory decision-making in doctor-patient discussions, a sequence of steps must be taken into account. Which step follows the model of participatory decision-making?” The five possible answers are “Say that a decision is pending”, “Inform about options” (correct answer), “Provide information about the advantages and disadvantages of the options”, “Inquire about understanding, thoughts and expectations” and “Determine preferences”.

The evaluation was carried out by dichotomising the five possible answers (correct/incorrect) and then determining a total value. According to the results of the Principal Axis Analysis reported below, five items from SS18 were used again in WS18/19.

#### 2.3. OSCE

The OSCE was checked on the basis of many years of experience with a communication OSCE in Mainz [3] derived from five stations that have already been developed (stations on the main topics of medical history, analysis of the stress reaction, checking and promoting compliance, participatory decision-making and notification of a cancer diagnosis according to the SPIKES protocol). The performance of the students was assessed by a total of 11 examiners over three examination days, using matching checklists that have already been developed and tested. The checklists related to the specific contents of the stations as well as aspects of the counselling techniques such as empathy, active listening, eye contact and open body position (for example “took an open body position”). The contents therefore correspond in the essential topics with the contents of the VSEs, although further topics and more theoretical knowledge were added. Performance was evaluated with a pre-determined weighting of the aspects of the checklists, with a maximum of 7 points for each station. An overall maximum of 35 points could therefore be achieved (absolute limit for a pass 60%).

#### 2.4. Statistical evaluation

The internal reliability of each examination was determined using Cronbach's Alpha. Spearman's rank correlation coefficient was used to examine the relationship between students' performance in the VSE and OSCE examinations. Kolmogorov-Smirnov tests were used to test the normal distribution of the variables. An iterative Principal Axis Analysis was performed on the VSE questions from each of the academic terms. Since the items under consideration are dichotomous variables that also show partly very low and very high item difficulties (see results section and table 2) and unequal marginal sum distributions are therefore present, odds ratios were calculated between all items. Odds ratios have values between zero and infinity and were transformed to values between -1 to +1 using the formula proposed by Yules. The iterative Principal Axis Analysis is based on the transformed odds ratios matrix. The Yules Y formula was applied with Y=(√OR-1)/(√OR+1) [28], [29], [30].

## 3. Results

The item characteristics for both VSEs are shown in table 2 (Tab. 2). The majority of items were answered correctly by most students (M=.75 SS 18 and M=.80 WS 18/19 across all means). The item-total correlation of the VSEs items ranged from -.04 to .44.

The criteria of Rost and Schermer were used to extract the number of relevant factors [31]. In the SS 18 group, 13 eigenvalues were greater than 1, whereas only one factor appeared to be plausible based on the scree plot. Only variables that had a communality of h²≥.16 and for which the absolute value was a≥.40 were represented by this factor (see table 3 (Tab. 3)). The remaining 8 items are difficult to represent accurately with a single factor. However, prioritising content plausibility over mathematical solution, merging 5 items (item 4, 11, 13, 16, 19) results in a significantly more appropriate content alignment under consideration of "patient-oriented, empathetic action and understanding based on the patient's situation and the content of the conversation" (Cronbach's α=.62, N=226). In the WS 18/19 group, 12 eigenvalues greater than 1 also resulted in only one factor based on the scree plot, whereas 14 items remained after applying the criteria. Here, 10 of the 14 items were also assigned to the aforementioned topic (3, 5, 7, 9, 12, 23, 24, 25, 26, 30; Cronbach's α=.60, N=193). Four further items show overlaps in content with the topic, but also with other topics, so that they could not be clearly assigned to the factor. Overall, it should be noted that the allocations and separations are not fully and finally accurate when considering the content of all items. Of the 5 items from the SS 18 group, 4 items were loaded again on the factor in the WS 18/19 group (items 23-26 WS 18/19).

The internal consistency of the VSE in the SS 18 group resulted in a Cronbach's Alpha coefficient of α=.55 (N=226) and in the WS 18/19 group of α=.62 (N=193). For the OSCE, there was an internal consistency of α=.60 (N=195).

The median of the VSE in SS18 was Mdn=23, IQR=3 for N=226, for the VSE in WS18/19 there was a Mdn=25, IQR=3 for N=192 and for the OSCE, a Mdn=26.25, IQR=2.75 also for N=192. For the WS18/19 group, the Spearman's rank correlation coefficient with respect to VSE and OSCE was r=.21 (p≤.01). The two variables were not normally distributed (Kolmogorov-Smirnov test OSCE D_192_=.09 and VSE WS 18/19 D_192_=.15, both p≤.01). Furthermore, an additional examination of the correlation of the items exclusively related to procedural knowledge from the VSE and OSCE results yielded a Spearman’s rank correlation coefficient of r=.25 (p≤.01). The variable “procedural knowledge” was also not normally distributed (Kolmogorov-Smirnov test: D_192_=.19, p≤.01).

The results of the evaluation of the VSEs are presented in table 4 (Tab. 4), with a majority of the students in favour of continuing the VSE and rating the practical relevance as relatively high. As the OSCE had already been tested and evaluated, we note that this was also rated as "good" in the WS 18/19 group with an overall score of Mdn=2, IQR=1 (N=145).

## 4. Discussion

We realised the goal of initial development and testing of a video-based e-exam for testing factual and procedural knowledge as components of competence in communication on the part of medical students in German-speaking countries. The implementation involved very complex technical processes, but went quite smoothly. As expected, there was a correlation between the result of the video exam and that of the practical OSCE exam, which was actually quite low. Consideration of the connection solely between the items regarding procedural knowledge in the VSE (according to the theoretical concept) and the OSCE turned out to be almost identical in terms of quantity, and it can be assumed that the wider range of topics in the VSE than in the OSCE can explain this result. Presumably, the variety of topics in the VSE overlaps with the classification according to competence levels (see also comments below). With regard to the complexity of the construct of communicative competence and the competence levels presented, it can also be assumed that, in addition to differences in the variety of topics, other areas or other competence levels (apart from factual and procedural knowledge) are also covered by communicative competence in the OSCE. Presumably this is also reflected in the level of the correlation. As expected, the majority of students rated the practical relevance of the examination with the use of videos higher than in the case of purely written examinations, and the doctor-patient consultations were experienced as realistic. Overall, these results suggest that the higher practical relevance that we desired was achieved by comparison with a purely text-based examination. However, it should be noted that the students had no experience with a purely written form of examination in this subject area (and little practical experience in the second semester), which is why their judgement can only be meaningful to a limited extent. A survey of experts would also be constructive in future. In addition, it should be noted that a comparison with a purely written examination was not implemented in order to avoid unreasonable stress for the students. However, such a comparison would be important in order to determine the greater practical relevance of the VSE than that of purely written examinations. Furthermore, the VSE does not yet adequately reflect the OSCE in its current form.

With regard to the test quality of the VSEs, we believe that the objectivity of their implementation and evaluation can be rated as very good (as expected), since the tasks and the conversation sequences in this test format are standardised and the evaluation is controlled with software. The objectivity can presumably be assessed as higher than that of the OSCE, in which the raters’ assessments often show slightly less agreement [17]. However, this assessment is based on plausibility and is not supported by data.

The internal consistencies of the two VSEs and the OSCE turned out to be low, although, contrary to expectations, the internal consistencies of the VSEs were little or no higher than those of the OSCE. This result should be interpreted in the context of the respective examination content. The OSCE that was used consisted of five stations with relatively narrowly defined subject areas. However, the VSEs were designed to cover a greater extent of the learning content, including theoretical concepts (see also Constructive Alignment [32]). Taking the entire course content into account, we found that 30 items are too few to achieve high internal consistency. An increase in the internal consistency of the VSEs could be achieved by increasing the number of items in the subject areas or, as with the OSCE, by reducing the number of subject areas. In accordance with Schecker [33], it seems important to decide whether to focus on bandwidth or specificity when constructing a test. He argued that in order to achieve a consistent scale, it is easier to include items from a single context, but with regard to teaching methodology, a wider range would be more desirable in a complex subject area. With regard to future video-based examinations, this question should therefore be asked and discussed again. According to Schecker, a lower value of internal consistency at the level of the two existing values of the VSEs is well tolerated if the didactic decision is in favour of bandwidth.

At this point, we should discuss the results of the study of the VSEs’ factor structure. In both tests, based on the statistics, there is at most a tendency towards one block of topics with regard to patient orientation and empathy, although many items do not count towards this factor and there is an overall overlap across all items. However, if one looks at the course content, this result is easy to understand. On the one hand, several models and fields of application of the course take into account the topics of patient orientation and empathy. On the other hand, according to the presentation of competence with medical communication as a complex construct, it is evident that different background knowledge and different aspects influence the items. It is plausible that some aspects relate to one another or are equally important in a task. If the results of the internal consistencies are combined with the results of the Principal Axis Analysis, a picture of mostly heterogeneous items is confirmed. A two-factor solution with factual knowledge and procedural knowledge was not in accordance with the results presented. It can be assumed that the effect of mostly heterogeneous subject areas is superimposed on a classification with regard to different levels of competence. The observation that in the VSE in WS18/19, significantly more items load one factor at a high level can probably be explained by the fact that the changed focus of the second exam was on procedural knowledge or tasks regarding the conduct of consultations, and thus reflects patient orientation/empathy. With regard to this point, it is worth reflecting critically on whether it is desirable to prioritise this topic in future exams. In general, it can be assumed that VSEs represent the learning content of the course and thus cover various aspects of communication competence (such as expressing empathy, informing, structuring, using the SPIKES protocol, etc.) as a correspondingly complex construct.

The statistical parameters also show that the VSEs were generally rather easy and the items show a low degree of item-total correlation. A goal for future exams of this kind will therefore be to increase their difficulty so that one can make a better differentiation when assessing skills.

The new form of examination was well received by the students, and the majority of the students supported continuation of the project. It must be noted, however, that the reasons for this have not been recorded and it is possible, for example, that the students have spoken out in favour of retaining the VSE compared to the OSCE due to lower emotional and time-related efforts of the VSE. This needs to be checked again.

While the development of the VSE was initially resource-intensive (as expected due to the large number of videos produced), examination material is now available for a large number of future exams. In our opinion, this means that the performance of the students can be checked with a VSE with fewer resources than with an OSCE (although this statement is not supported by data).

## 5. Conclusion

Finally, if in practice one needs to carry out a test based on the observable level of action, the OSCE still comes closest to the conditions in a real doctor-patient consultation. If an examination needs higher practical relevance than a purely written examination, a video-based examination may well be a suitable form of examination that is more resource-efficient in the medium and long term than an OSCE, and can probably achieve a higher level of objectivity. However, these conclusions are based partly on plausibility (such as resource expenditure and objectivity) and are not supported by data. The internal consistency for both tests may also be influenced by the range of topics and the number of items or stations, while an increase in internal consistency appears to be easier to implement with the VSE than with the OSCE. This also means that it is easier to cover more learning objectives comprehensively with a VSE.

The newly designed video-based e-exam is therefore a very promising instrument for testing certain aspects of the communication competency of medical students, subject to the above-mentioned requirements and limitations. However, there is still a need for improvement in the difficulty of the items, critical reflection on the level of internal consistency, and direct comparison with a purely written examination. Further experience and research at other universities, as well as the development and testing of modified concepts seems necessary and worthwhile with regard to the above-mentioned limitations.

## Notes

The hypotheses and results are based on the dissertation of Ms. Stephanie Ludwig.

## Acknowledgements

We thank the BMBF for funding the project. We would also like to thank Prof. Wermuth, Prof. Hardt and Prof. Beutel, as well as Dr. Schappert, Dr. Ditter and Dr. Seifert, all examiners, simulated patients, scientific assistants, the learning clinic with Mr. Thomas Nowak, the media centre, the ZDV and other participants for their support in implementing this project.

## Funding

This work was supported by the Federal Ministry of Education and Research (Grant number 16DHL1032).

## Profiles

**Location:** Mainz University Medical Center

**Subject/ professional group: **Human medicine

**Number of students per semester: **approx. 200

**Has a longitudinal communication curriculum been implemented?** A pilot implementation is currently underway as part of the LONGKOM project, [https://www.unimedizin-mainz.de/lernklinik/startseite/projekte/longkom-kommunikative-kompetenzen-von-aerztinnen-und-aerzten.html]

**In which semesters are communicative and social skills taught? **2^nd^ and 5^th^ semesters, possibly more, currently recorded as part of the LONGKOM project

**Which teaching formats are used? **Role play, simulated patient interviews, lecture, blended learning, sample videos

**In which semesters are communicative and social skills tested (formative or summative and/or graded)? **2^nd^, 5^th^, 9^th^ semester

**Which examination formats are used? **OSCE (summative), video-based single-choice exam (summative), video-based e-exercise (formative), essay exam

**Who (e.g. clinic, institution) is entrusted with development and implementation? **Clinic and Polyclinic for Psychosomatic Medicine and Psychotherapy, Department of Medical Psychology and Medical Sociology, Rudolf Frey Learning Clinic Mainz and practice of Dr. B. Schappert, Mainz

## Current professional roles of the authors

- Dipl.-Psych. Stephanie Ludwig: Research assistant (Clinic and Polyclinic for Psychosomatic Medicine and Psychotherapy, University Medical Center Mainz) in the BMBF-funded ‘digiRole’ project. Also active as a licensed psychological psychotherapist.
- Lina Behling, MA: Lina Behling is a research assistant at the Center for Quality Assurance and Development at Johannes Gutenberg University, Mainz.
- Univ.-Prof. Dr. Uwe Schmidt: Uwe Schmidt is Professor of University Research at the Institute for Sociology at Johannes Gutenberg University Mainz, and Head of the Center for Quality Assurance and Development. His research focuses on empirical university research and evaluation research.
- Dr. rer. physiol. Dipl.-Psych. Sabine Fischbeck, MME: Research assistant and teaching officer for medical psychology and medical sociology in Mainz, Master of Medical Education and psycho-oncologist.

## Competing interests

The authors declare that they have no competing interests. 

## Figures and Tables

**Table 1:**
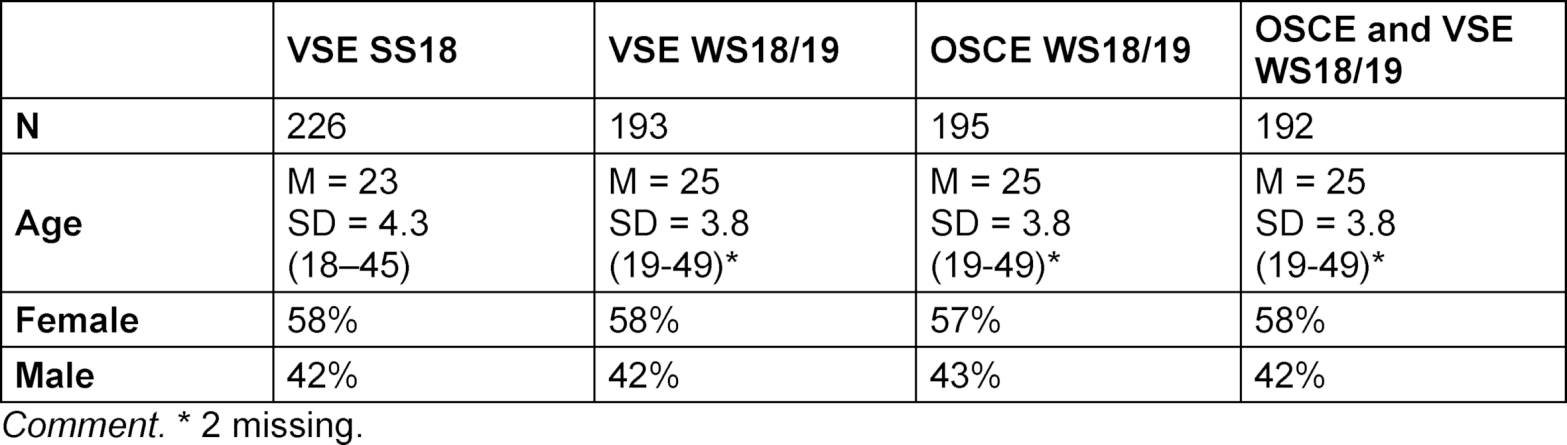
Study participants

**Table 2:**
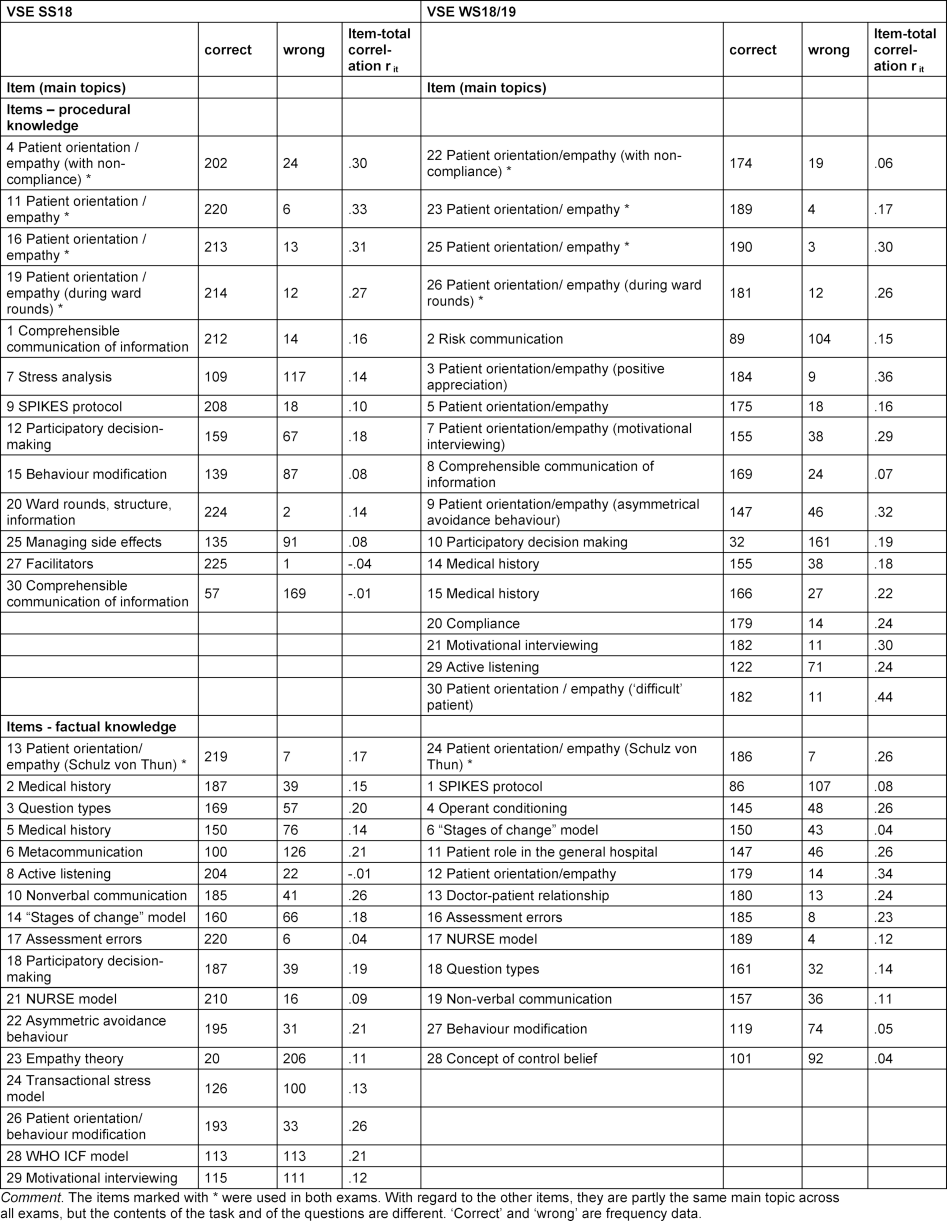
Item characteristics of the VSEs in SS18 (N=226) and in WS18/19 (N=193)

**Table 3:**
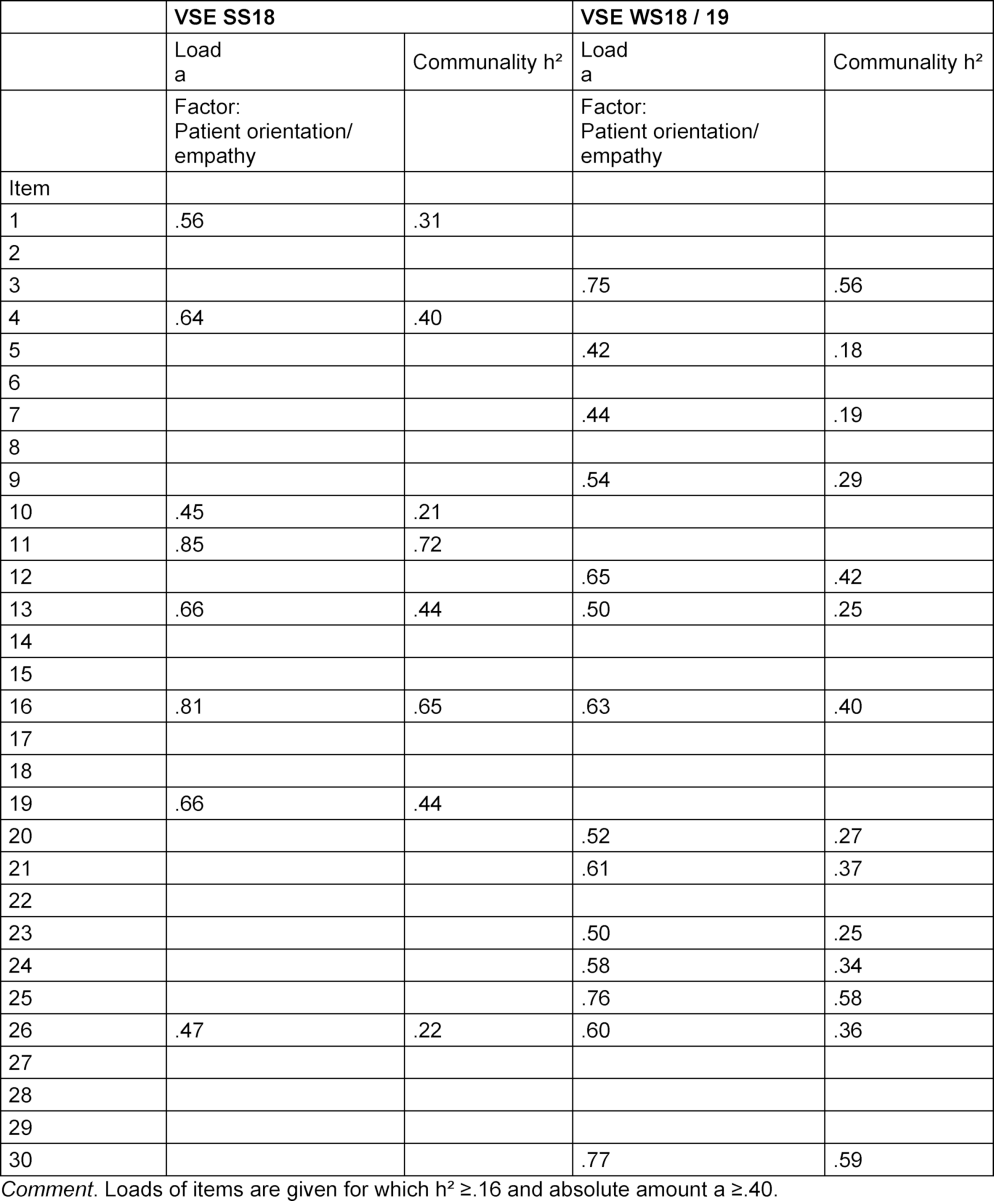
Principal axis analyses of the VSEs in SS18 and WS18/19, single factor solutions

**Table 4:**
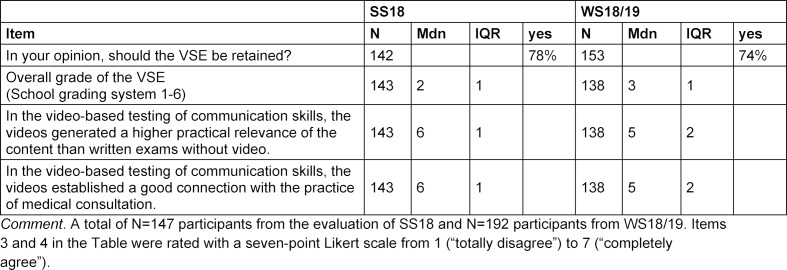
Evaluation of results

## References

[1] Härtl A, Bachmann C, Blum K, Höfer S, Peters T, Preusche I, Raski B, Rüttermann S, Wagner-Menghin M, Wünsch A, Kiessling C, GMA-Ausschuss Kommunikative und Soziale Kompetenzen (2015). Desire and reality--teaching and assessing communicative competencies in undergraduate medical education in german-speaking europe-a survey. GMS Z Med Ausbild.

[2] Patterson F, Ashworth V, Zibarras L, Coan P, Kerrin M, O'Neill P (2012). Evaluations of situational judgement tests to assess non-academic attributes in selection. Med Educ.

[3] Fischbeck S, Mauch M, Leschnik E, Beutel ME, Laubach W (2011). Überprüfung ärztlicher kommunikativer Kompetenz mittels einer OSCE bei Studierenden der Medizin im ersten Studienjahr. Psychother Psychosom Med Psychol.

[4] Bundesministerium für Gesundheit (2017). Beschlusstext zum "Masterplan Medizinstudium 2020".

[5] Association of American Medical Colleges (1998). Report I. Learning Objectives for Medical Student Education. Guidelines for Medical Schools.

[6] UK Foundation Programme Office (2016). The Foundation Programme Curriculum 2016.

[7] Aspegren K (1999). Beme guide no. 2: Teaching and learning communication skills in medicine-a review with quality grading of articles. Med Teach.

[8] Baribeau DA, Mukovozov I, Sabljic T, Eva KW, deLottinville CB (2012). Using an objective structured video exam to identify differential understanding of aspects of communication skills. Med Teach.

[9] Laidlaw A, Salisbury H, Doherty EM, Wiskin C (2014). National survey of clinical communication assessment in medical education in the united kingdom (UK). BMC Med Educ.

[10] Chong L, Taylor S, Haywood M, Adelstein BA, Shulruf B (2018). Examiner seniority and experience are associated with bias when scoring communication, but not examination, skills in objective structured clinical examinations in australia. J Educ Eval Health Prof.

[11] Daniels VJ, Harley D (2017). The effect on reliability and sensitivity to level of training of combining analytic and holistic rating scales for assessing communication skills in an internal medicine resident osce. Patient Educ Couns.

[12] Dong T, LaRochelle JS, Durning SJ, Saguil A, Swygert K, Artino AR (2015). Longitudinal effects of medical students' communication skills on future performance. Mil Med.

[13] Miller GE (1990). The assessment of clinical skills/competence/performance. Acad Med.

[14] Kopp V, Möltner A, Fischer MR (2006). Key-Feature-Probleme zum Prüfen von prozeduralem Wissen: Ein Praxisleitfaden. GMS Z Med Ausbild.

[15] Schubert S, Ortwein H, Dumitsch A, Schwantes U, Wilhelm O, Kiessling C (2008). A situational judgement test of professional behaviour: Development and validation. Med Teach.

[16] Koczwara A, Patterson F, Zibarras L, Kerrin M, Irish B, Wilkinson M (2012). Evaluating cognitive ability, knowledge tests and situational judgement tests for postgraduate selection. Med Educ.

[17] Chenot JF, Ehrhardt M (2003). Objective structured clinical examination (OSCE) in der medizinischen Ausbildung: Eine Alternative zur Klausur. Z Allg Med.

[18] Schmitz FM, Schnabel KP, Bauer D, Bachmann C, Woermann U, Guttormsen S (2018). The learning effects of different presentations of worked examples on medical students' breaking-bad-news skills: A randomized and blinded field trial. Patient Educ Couns.

[19] Humphris GM, Kaney S (2000). The objective structured video exam for assessment of communication skills. Med Educ.

[20] Hulsman RL, Mollema ED, Oort FJ, Hoos AM, de Haes JC (2006). Using standardized video cases for assessment of medical communication skills: Reliability of an objective structured video examination by computer. Patient Educ Couns.

[21] Fischbeck S, Mauch M, Unterrainer J, Berth H (2013). Vom Kompetenzwissen zum praktischen Können: Arzt-Patient-Kommunikation prüfen mit Key-Feature-Klausuren und OSCE. In balance. Abstracts zur Jahrestagung der Deutschen Gesellschaft für Medizinische Psychologie 2013.

[22] Kurtz S, Silverman J, Draper J (2006). Teaching and learning communication skills in medicine.

[23] Back AL, Arnold RM, Baile WF, Fryer-Edwards KA, Alexander SC, Barley GE, Gooley TA, Tulsky JA (2007). Efficacy of communication skills training for giving bad news and discussing transitions to palliative care. Arch Intern Med.

[24] Kanfer FH, Reinecker H, Schmelzer D (2012). Selbstmanagement-Therapie.

[25] Hamann J, Loh A, Kasper J, Neuner B, Spies C, Kissling W, Härter M, Heesen C (2006). Partizipative Entscheidungsfindung. Implikationen des "shared decision making" für Psychiatrie und Neurologie. Nervenarzt.

[26] Baile WF, Buckman R, Lenzi R, Glober G, Beale EA, Kudelka AP (2000). Spikes-a six-step protocol for delivering bad news: Application to the patient with cancer. Oncologist.

[27] Hoppe-Seyler T, Gartmeier M, Möller G, Bauer J, Wiesbeck A, Karsten G (2014). Entwicklung von Lehrfilmen zur Gesprächsführung zwischen Realitätsnähe und systematischer didaktischer Gestaltung. ZFHE.

[28] Edwards AW (1963). The Measure of Association in a 2 × 2 Table. J Royal Stat Soc.

[29] Leonhart R (2009). Lehrbuch Statistik.

[30] Wermuth N (2019). How can graphical Markov models aid causal inference?. DAGStat 2019. Statistics under one umbrella. Conference Guide. München, 18.-20. März 2019.

[31] Rost DH, Schermer FJ (1986). Strategien der Prüfungsangstverarbeitung. Z Diff D P.

[32] Biggs J (1996). Enhancing teaching through constructive alignment. High Educ.

[33] Schecker H, Krüger D, Parchmann I, Schecker H (2014). Überprüfung der Konsistenz von Itemgruppen mit Cronbachs alpha. Methoden in der naturwissenschaftsdidaktischen Forschung.

